# Evaluating the mitochondrial genomic diversity, global distribution and niche overlap of two invasive *Phthorimaea* species

**DOI:** 10.1016/j.heliyon.2024.e29010

**Published:** 2024-04-04

**Authors:** Inusa Jacob Ajene, Helen Heya, Fathiya Mbarak Khamis

**Affiliations:** a International Center of Insect Physiology and Ecology, Nairobi, Kenya; b Kenya Plant Health Inspectorate Service, Nairobi, Kenya

**Keywords:** Gelechiidae, Habitat suitability, Mitochondrial diversity, *Phthorimaea*, Solanaceae, Species distribution

## Abstract

This study sought to evaluate the genetic diversity of two invasive *Phthorimaea* species (*Phthorimaea operculella* Zeller and *Phthorimaea absoluta* Meyrick), and identify potential niche overlap of both species. The complete mitogenomes of *P. operculella* and *P. absoluta* were sequenced and compared. Furthermore, the diversity within the family Gelechiidae was assessed. Subsequently, two species distribution models (MaxEnt and BIOCLIM) were used to identify niche overlaps of both species globally. The complete mitogenomes of both species were similar in size and structure, with a pairwise identity of 92.3%. The models showed a niche overlap of both species and revealed areas of marginal to high suitability for both pests in countries where they have not been reported. Therefore, these results present a compelling case for a deeper genetic and ecological investigation of the Gelechiidae family for taxonomic harmonization, an early warning for surveillance, stricter phytosanitary considerations and preventive management against the spread of the pests.

## 1 Introduction

Solanaceous vegetable plants include some of the world's most economically important food crops, with crops such as potato, tomato, eggplant, and pepper representing 39% of horticultural production in the world [1]. Potato (*Solanum tuberosum* L.) and tomato (*Solanum lycopersicum* L.) are major staples in diets worldwide. Potato is the fourth major food crop around the world [2]. Also, tomato is the third largest solanaceous crop after potato and sweet potato (*Ipomea batatas* (L.) Lam) and first among all vegetables. With 187 M tonnes produced globally in 2020, it represents 17% of all vegetables grown worldwide [3]. The production of both crops is severely hampered by numerous factors, including pests and diseases. Important arthropod pests include thrips (*Frankliniella* spp. Karny 1910), potato psyllids (*Bactericera cockerelli* Šulc 1909), red leafminers (*Liriomyza* spp. Mik 1894) leafhoppers, beetles (*Leptinotarsa decemlineata* Say 1824), aphids (*Myzus persicae* Sülzer 1776), and moths (*Tecia solanivora* Povolný 1973*, Phthorimaea operculella* Zeller 1989, *Keiferia lycopersicella* Walsingham 1987*, Phthorimaea absoluta* Meyrick 1917 and *Symmetrischema tangolias* Gyen 1913) [2,[4], [5], [6]]. The potato tuber moth, *Phthorimaea operculella* Zeller and the tomato leaf miner *Phthorimaea absoluta* Meyrick belonging to the moth family Gelechiidae are serious pests of potatoes and tomatoes, respectively. *Phthorimaea operculella* has been reported to be the most significant insect pest of potatoes in storage and field in North Africa, Asia, and the Middle East [7,8], while *P. absoluta* is currently the dominant insect pest of tomatoes causing significant production losses in all the invaded regions [6]. *Phthorimaea absoluta* is a close relative of *P. operculella* and can sometimes be taxonomically confused with this pest [2,9,10]. Tomato is the main host plant of *P*. *absoluta*, although the pest can feed, develop and reproduce on potato [11]. Similarly, the principal host plant of *P. operculella* is potato, but the pest can complete its lifecycle on tomato and other solanaceous plants [4]. Furthermore, there is an ambiguous taxonomy among some Gelechiid species. For example, *S. tangolias* has previously been classified under the *Phthorimaea* genus as *Phthorimaea aquilina* Meyrick, 1917, *Phthorimaea melanoplintha* Meyrick, 1925, *Phthorimaea plaesiosema* Turner and *Phthorimaea tuberosella* Busck. Similarly, *K. lycopersicella* has previously been named *Phthorimaea elmorei* and *Phthorimaea lycopersicella*. The taxonomy of *P. absoluta* has also changed numerous times [[12], [13], [14], [15]]. However, these changes in nomenclature were made based on the characteristics of male and female genitalia [16]. After numerous taxonomic changes, the genus *Tuta* was recognized as a valid genus and formal combination of *Tuta absoluta* (Meyrick) was published by Povolný [17]. The species was recently reclassified into the *Phthorimaea* genus using cladistic parsimony analysis of 22 morphological characters from members within the genera *Phthorimaea*, *Scrobipalpuloides* and *Tuta* [16]. Although *P. operculella* is commonly referred to as the potato tuberworm [18], *Tecia solanivora* (Povolný) and *Symmetrischema tangolias* (Gyen) are also known as potato tuber worms. Additionally, *Symmetrischema tangolias* is also known as the tomato stemborer. Therefore, taxonomic ambiguity of this genus needs to be further resolved through deployment of stringent molecular markers and morphological taxonomic tools for proper management and modelling of their distribution and risk of spread. These 'tuber worms' (*Tecia solanivora* and *Symmetrischema tangolias*) are geographically restricted to South and Central America, Australia and the Philippines [19,20]. Therefore, evaluating the genetic variations, phylogeny, and spatial distribution of these closely related species will provide insight into the biology of these species and their potential habitat expansion. Furthermore, investigating the distribution and habitat range of these pests is critical because changes in the habitat of pests severely damage local biodiversity and agriculture [[21], [22], [23]]. Thus, species distribution models and softwares such as BIOCLIM, CLIMEX, MaxEnt, Support Vector Machine and Boosted Regression Trees are essential tools for evaluating the potential distribution of species [24,25]. These models are used to assess possible changes in species' geographic ranges [26,27] and aid in biodiversity conservation [28]. Currently, *P. operculella* has been reported in most potato production areas in Asia, Europe, the Americas, Australia and Africa [[29], [30], [31], [32]]. In comparison, *P. absoluta* has been reported only in Africa, Asia, Europe, South America and Central America [6]. Individually, the distribution and potential spread of these pests have been assessed in the tropics [6,33,34]. Nevertheless, the niche overlap of both species has not been researched and while *P. operculella* has been reported on almost every continent worldwide, *P. absoluta* continues to spread at an alarming rate and is expanding its host range by attacking other solanaceous crops that are important sources of food and income [6]. Therefore, identifying the habitat suitability of both species, their potential distribution and regions of suitability where either species has not invaded will provide key information required for analysing the risk of pest invasions and identifying management spots [35,36]. Considering the economic importance of *P. operculella* and *P. absoluta* to potato and tomato production and trade, predicting their risk of spread is critical for early warning of invasions and developing sustainable pest management systems.

Studying the mitochondrial DNA (mtDNA) has various advantages in species identification, such as a lack of sequence obscurity from dissimilar genotypes and rates of mutation [37]. Furthermore, in-depth phylogenomics is necessary for resolving the taxonomic ambiguity of the species within the Gelechiidae family, as prior classification and reclassification have been based on their morphology [16]. Furthermore, these pests cause significant economic losses and to essential food crops (potato and tomato) and the production losses of crops due to these pests are on the rise. Thus, understanding the biology of the pest and species distribution modelling can provide experts with critical information required to formulate management and quarantine strategies [38]. Therefore, in this study, we sought to establish a baseline for investigating the genetic diversity, niche overlap and distribution of *Phthorimaea* species, as there is an established overlap in the host range of the genera by comparatively assessing the complete mitochondrial genomes of the two species, modelling the distribution and predicting their potential distribution to identify areas of spatiotemporal risk to inform phytosanitary policies for early warning and implementation of preventative and management strategies.

## 2 Methods

### 2.1 Sample collection and DNA extraction

*Phthorimaea absoluta was* obtained from tomato plants in Naivasha (S00°40′05.6″E036°23′09.1″) and Taita Taveta (S03°22′52.1″E037°43′10.7″) in Kenya and *P. operculella* was obtained from potato tubers in Limuru (S01°14′28.7″E036°44′47.8″), Kenya and stored in 96% ethanol. One adult specimen representative of the species was imaged and deposited in the Biosystematics unit of the International Center of Insect Physiology and Ecology (*icipe*), Nairobi, Kenya. Two specimens per species (*P. absoluta* and *P. operculella*) were randomly selected for next-generation sequencing (NGS). Total DNA was individually extracted from each insect using the Isolate II Genomic DNA Kit (Bioline, London, UK). DNA extracts were stored at −20 °C until further analyses.

### 2.2 Mitogenome sequencing, assembly and annotation

The complete mitochondrial genome of two specimens of *P. operculella* (PO-KE) and *P. absoluta* (PA-KE) was sequenced to assess the genetic relationship among the species. Two specimens per species were used for NGS to recover complete mitochondrial genome sequences. Total DNA from each sample was sequenced separately using the DNBseq sequencing platform at BGI Genomics (BGI, Tai Po, N.T, Hong Kong). Mapping and assembly of the new mitogenomes were done using SPAdes v.3.13.0 [39], and the resulting contigs were identified by BLAST + [40,41]. Subsequently, each mitogenome was mapped using publicly available mitogenome sequences of *P. opercullela* (MW540822) and *P. absoluta* (NC_050874) as a reference sequence using Geneious Prime v2019.1 [42] for confirmation. Open reading frames of Protein Coding Genes (PCGs) were identified in Geneious, using invertebrate mitochondrial genetic code. Transfer RNAs (tRNAs) were identified using ARWEN software [43]. Manual counting of the intergenic spacers (IGS) and overlapping regions was done. The mitogenome sequences were deposited in GenBank under the BioProject: PRJNA902348 (https://www.ncbi.nlm.nih.gov/bioproject/PRJNA902348).

## 3 Comparison of mitogenome sequences

To assess genetic divergence and identify single nucleotide polymorphisms (SNPs) within the PCGs among the mitogenomes, multiple sequence alignments of the sequences obtained in this study and five (5) publicly available sequences of members of the family Gelechiidae (Table S1) were performed using the MAFFT algorithm [44] available in Geneious Prime. Genetic distances among all sequences were calculated using nucleotide pairwise distances (p-distances) in MEGA v. 11.0.9 [45] under the Kimura 2-parameter model [46].

Phylogenetic relationships among members of the family Gelechiidae were reconstructed using the new *Phthorimaea* mitogenomes generated in this study, along with the five complete mitogenomes available on GenBank for the family Gelechiidae (as of November 2022), with *Spodoptera frugiperda* as an outgroup (See Table S1 in supporting information). The Sequences of 13 protein-coding genes were extracted based on annotations. All sequences were concatenated and aligned using the MAFFT algorithm in Geneious Prime. The evolutionary model was selected using jModelTest2 [47]. The ML method was implemented in PhyML [48] to construct the phylogenetic tree. Nodal support was based on bootstrap analysis with 100 replicates.

### 3.1 Species phylogeny

To obtain insights into the phylogeny of the two *Phthorimaea* species, publicly available datasets of COI sequences were used to provide a broader context to the new sequences (Table S2). Multiple sequence alignment, involving a total of 322 sequences (including an extraction of the COI gene sequences from the mitogenomes generated in this study and of 320 publicly available sequences), was performed using the MAFFT algorithm available on Geneious Prime. Genetic divergences among the species were calculated as pairwise distances (p-distances) in MEGA v. 11.0.9 under the Kimura 2-parameter model (K2P). A maximum-likelihood (ML) tree was also constructed to show the phylogenetic relationships among the sequences, using one representative sequence for each Gelechiid species. The ML tree was generated in MEGA v. 11.0.9 with 1000 bootstrap replicates. Inter-specific genetic distances were represented with multidimensional scaling analysis using the 'cmdscale' function in R version 3.5.1 [49] on the genetic distance matrix to generate the plot for principal coordinate analysis (PCoA).

## 4 Habitat suitability modelling

### 4.1 Species data

**Fig. 1:**
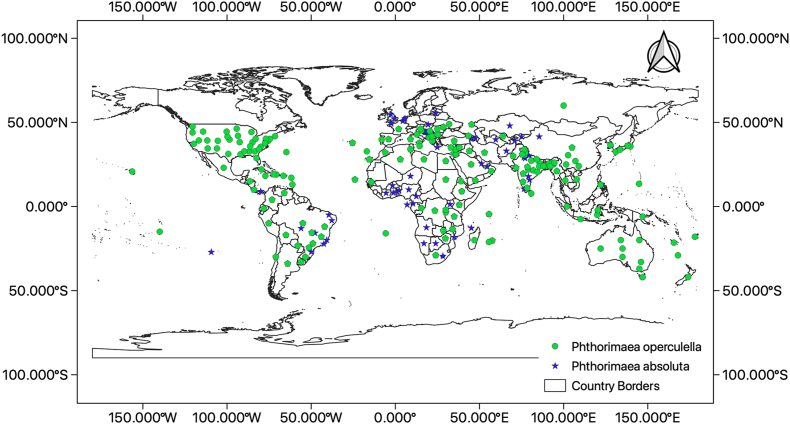
Global distribution of *Phthorimaea operculella* and *Phthorimaea absoluta* as obtained from Centre for Agriculture and Bioscience International (CABI) Invasive Species Compendium (CABI, 2022).

Global presence points from reports of *P. operculella* and *P. absoluta* were obtained from the Centre for Agriculture and Bioscience International (CABI) Invasive Species Compendium [50,51], European and Mediterranean Plant Protection Organization (EPPO) [52,53] and Global Biodiversity Information Facility (GBIF) [54,55] (Fig. 1). Duplicate coordinates were removed from the final dataset prior to the model runs. Models were run individually for each species, and the resulting models were overlapped to obtain a consensus prediction for regions of niche overlap for both species.

### 4.2 Models

Two species distribution models (Maximum Entropy and BIOCLIM) were selected to study the potential distribution of the two *Phthorimaea* species globally; (a) The classic climate-envelope-model BIOCLIM [56] and (b) Maximum Entropy (MaxEnt), which is a general-purpose method for making predictions from incomplete information [57]. These models were chosen to represent a range of model complexity and compensate for the restrictions of the individual models through the consensus approach [58].

### 4.3 Selection of bioclimatic variables

The environmental variables used in this study included 19 bioclimatic predictors (Table S3) of 2.5 km spatial resolution using the baseline average [1950–2000] [59]. The environmental variables were obtained from the WorldClim database (http://www.worldclim.org/). A Jacknife test for variable importance was run on the selected variables in Maxent to determine each variable's percentage contribution to the model (see Fig. S1).

### 4.4 Model evaluations

The accuracy of the model was evaluated using the correlation coefficient and the area under the receiver operating characteristic curve (AUC curve) [60]. The AUC represents the relation between the false positive fraction and the sensitivity for several thresholds [57,61]. All models were run in R v3.5.1 environment via R-Studio, the results were exported as ASCII files and visualized with QGIS software v2.18.15 [62].

### 4.5 Model calibration

The MaxEnt model was calibrated by comparing the presence locations for *P. absoluta* and *P. operculella* (occurrence data) against pseudo-absence points generated automatically in MaxEnt by random selection from all points within the studied area, excluding available presence points [63]. The model was trained using 75 % of the presence data and validated using 25 %. The model was run with 5000 iterations and >10,000 background points. For the Bioclim model, the standard deviation and mean for each environmental variable were used to calculate bioclimatic envelopes. The level of fitness between the environmental values on a point classifies each point as Suitable, Marginal, or Unsuitable for presence. The presence data were compared against 10,000 background points randomly generated from all points outside of the suitable area [63,64].

### 4.6 Model consensus and niche overlap

A two-model consensus of the Maxent and BIOCLIM models was used because single models with high accuracy may not be efficient in projecting onto new areas or climate conditions [65]. Thus, a combination of multiple models will make up for the deficiencies of one model. To get a two-model consensus, the raster outputs from the individual models were rescaled to uniform values between 0 and 1, then weighted based on their AUC scores and combined. Finally, a raster stack of the predictions was made, and the average computed. The model outputs were classified based on the ecoclimatic index (EI) where; EI = 0 (unsuitable), EI ≤ 0.19 (marginally suitable), EI = 0.2–0.49 (Highly suitable) and EI ≥ 0.5 (optimally suitable). The niche overlap was determined using the raster calculator in QGIS to resolve the current habitat overlaps between the two species. Three metrics were considered: niche expansion (climatic conditions available in both native and invasive ranges but only occupied in the invaded range), niche unfilling (climatic conditions that are available, but not yet occupied in the invaded range) and niche stability conditions filled in both native and invaded ranges). The overlap was calculated by subtracting the individual species model raster outputs from the consensus model raster output to exclude the areas of environmental suitability for individual species, and thus remaining with areas of suitability shared by both species [58,66].

## 5 Results

### 5.1 General features of the *Phthorimaea* mitogenomes

**Fig. 2:**
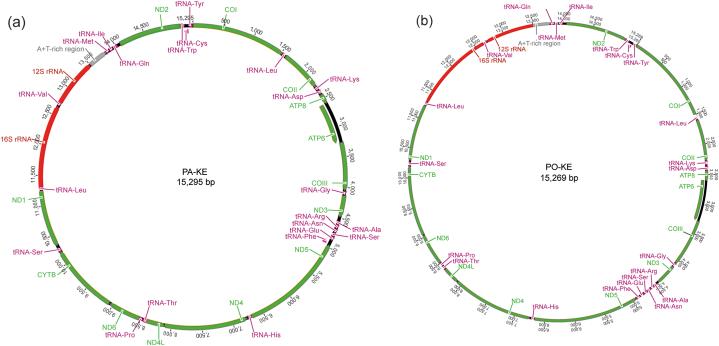
Circular map of the mitochondrial genome of (a) *Phthorimaea absoluta* (PA-KE) and (b) *Phthorimaea operculella* (PO-KE). Protein-coding genes, transfer RNAs and ribosomal RNAs are represented with standard abbreviations.

The mitogenomes of both species (*P. operculella* and *P. absoluta*) were identical in the main features. They had the typical Metazoan complement of 13 Protein Coding Genes (PCGs), two ribosomal RNA (rRNA) genes, 22 transfer RNA (tRNA) genes and an AT-rich non-coding region (Fig. 2). The complete sequences for *P. absoluta* had a total size of 15,295 bp (Fig. 2a), and *P. operculella* had a total length of 15269 bp (Fig. 2b), which is similar to the average for the complete mitogenome of other the Gelechiids (15,255 bp). The gene order was identical to the other species in the family Gelechiidae, which was also identical to the hypothesised ancestral mitogenome organisation in insects [67]. Twenty-six genes were located on the majority strand (J-strand), and the other 12 genes were on the minority strand (N-strand).

### 5.2 Protein-coding genes

The combined length of the 13 PCGs was 11,206 bp and 11,193 bp for *P. absoluta* and *P. operculella,* respectively. The longest PCG was ND5 in both species (*P. absoluta*; 1735 bp and *P. operculella*; 1732 bp), while the shortest PCG was ATP8 (*P. absoluta*; 171 bp and *P. operculella*;165 bp) (Table 1). These features were identical to the mitogenome features of other members of the Gelechiidae family, which had an average PCG length of 10,801, with ND5 and ATP8 being the longest and shortest PCGs, respectively.

**Table 1:** Mitogenome features of the complete sequence of *Phthorimaea absoluta* and *Phthorimaea operculella* specimens. J – majority strand; N – minority strand. IGS – intergenic regions, with negative values representing overlapping regions.

Region			*P. absoluta*					*P. operculella*				
	Code	Strand	Start	Stop	Coordinates	Size (bp)	IGS	Start	Stop	Coordinates	Size (bp)	IGS
COI	–	J	CGA	TAA	1–1536	1536	3	CGA	TAA	1–1536	1536	2
tRNA-Leu	L2	J	–	–	1532–1599	68	−5			1532–1599	68	−5
COII	–	J	ATG	CTT	1600–2281	682	–	ATG	CTT	1600–2281	682	–
tRNA-Lys	K	J	–	–	2282–2352	71	8			2282–2352	71	21
tRNA-Asp	D	J	–	–	2361–2428	68	–			2374–2443	70	–
ATP8	–	J	–	–	2429–2599	171	−7			2444–2608	165	−7
ATP6	–	J	ATG	TAA	2593–3270	678	–	ATC	TAA	2602–3279	678	−1
COIII	–	J	ATG	TAA	3271–4059	789	2	ATG	TAA	3279–4067	789	2
tRNA-Gly	G	J	–	–	4062–4128	67	–			4070–4136	67	–
ND3	–	J	ATT	TAA	4129–4482	354	69	ATT	TAA	4137–4490	354	29
tRNA-Ala	A	J	–	–	4552–4622	71	–			4520–4586	67	–
tRNA-Arg	R	J	–	–	4623–4686	64	2			4587–4652	66	2
tRNA-Asn	N	J	–	–	4689–4754	66	–			4655–4720	66	–
tRNA-Ser	S1	J	–	–	4757–4822	66	−2			4721–4786	66	4
tRNA-Glu	E	J	–	–	4823–4888	66	–			4791–4858	68	6
tRNA-Phe	F	N	–	–	4887–4952	66	–			4865–4932	68	–
ND5	–	N	ATA	T-	4953–6687	1735	–	AAT	T-	4933–6664	1732	–
tRNA-His	H	N	–	–	6688–6753	66	7			6665–6730	66	8
ND4	–	N	CAT	TTA	6761–8101	1341	1	CAT	TTA	6739–8079	1341	1
ND4L	–	N	CAT	TTA	8103–8396	294	2	AAT	TTA	8081–8368	288	8
tRNA-Thr	T	J	–	–	8399–8463	65	–			8377–8440	64	–
tRNA-Pro	P	N	–	–	8464–8528	65	2			8441–8505	65	2
ND6	–	J	ATA	TAA	8531–9061	531	9	ATA	TAA	8508–9038	531	7
CYTB	–	J	ATG	TAA	9071–10219	1149	7	ATG	TAA	9046–10194	1149	2
tRNA-Ser	S2	J	–	–	10227–10293	67	22			10197–10263	67	18
ND1	–	N	CAT	TAA	10316–11249	934	1	CAT	TTA	10282–11220	939	1
tRNA-Leu	L1	N	–	–	11251–11318	68	–			11222–11289	68	–
16s rRNA	–	J	–	–	11319–12662	1344	–			11290–12630	1341	–
tRNA-Val	V	N	–	–	12663–12728	66	–			12631–12696	66	–
12S rRNA	–	J	–	–	12729–13504	776	–			12697–13504	808	–
AT-rich region	–	J	–	–	13505–13824	320	–			13505–13792	288	–
tRNA-Met	I	J	–	–	13825–13893	69	–			13793–13861	69	–
tRNA-Ile	Q	J	–	–	13894–13958	65	1			13862–13926	65	2
tRNA-Gln	M	N	–	–	13960–14028	69	56			13929–13997	69	57
ND2	–	J	ATT	TAA	14085–15098	1014	−2	ATT	TAA	14055–15068	1014	1
tRNA-Trp	W	J	–	–	15100–15164	65	−8			15070–15137	68	8
tRNA-Cys	C	N	–	–	15157–15222	66	4			15130–15197	68	3
tRNA-Tyr	Y	N	–	–	15227–15292	66	3			15201–15267	67	2

### 5.3 tRNAs and rRNAs

For both species, the large ribosomal RNA gene (1344 bp and 1341 bp for *P. absoluta* and *P. operculella,* respectively) was located between tRNA^Leu1^ and tRNA^Val^. The small ribosomal RNA gene (776 bp and 808 bp for *P. absoluta* and *P. operculella,* respectively) was situated between tRNA^Val^ and the AT-rich region. The tRNA sizes varied between 64 bp (tRNA^Arg^) and 71 bp (tRNA^Lys^ and tRNA^Ala^) for *P. absoluta* and 64 bp (tRNA^Thr^) and 71 bp (tRNA^Lys^) for *P. operculella* (Table 1).

### 5.4 Non-coding AT-rich, intergenic and overlapping regions

*Phthorimaea absoluta* had four (4) short gene overlaps, mostly involving tRNAs (maximum overlap = 7 bp), while *P. operculella* had two (2) short gene overlaps. Intergenic regions were found at 17 locations in *P. absoluta*, while intergenic spacers (IGS) were found at 21 locations in *P*. *operculella*. Furthermore, the largest intergenic spacer identified in *P. absoluta* was between tRNA^Gly^ and ND3 (69 bp), while in *P*. *operculella,* it was between tRNA^Ile^ and ND2 (57 bp) (Table 1).

### 5.5 Mitogenomes comparison between *P. operculella* and *P. absoluta*

**Fig. 3:**
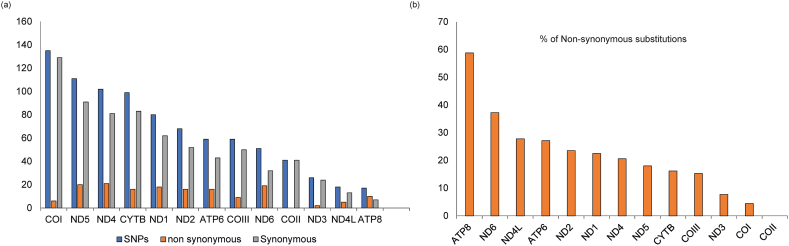
Comparison of the mitochondrial sequences of *Phthorimaea absoluta* and *Phthorimaea operculella* specimens based on the protein-coding genes. The differences are represented as (a) the number of single nucleotide polymorphisms and (b) the percentage of non-synonymous substitutions relative to the size of each protein-coding gene.

The alignment of the complete mitogenomes of *P. absoluta* and *P. operculella* had 14,169 bp identical sites with a pairwise identity of 92.3%. Between *P. absoluta* and *P. operculella*, there were 866 SNPs across the PCGs, with the highest number of SNPs (135) observed in COI and the least observed in ATP8 (17) (Fig. 3a). The 866 SNPs resulted in 158 non-synonymous substitutions, with the highest percentage of non-synonymous substitutions seen in ATP8 (59%) and the least was in COI (4%). There were no non-synonymous substitutions in COII (Fig. 3b).

### 5.6 Phylogeny of the family gelechiidae

**Fig. 4:**
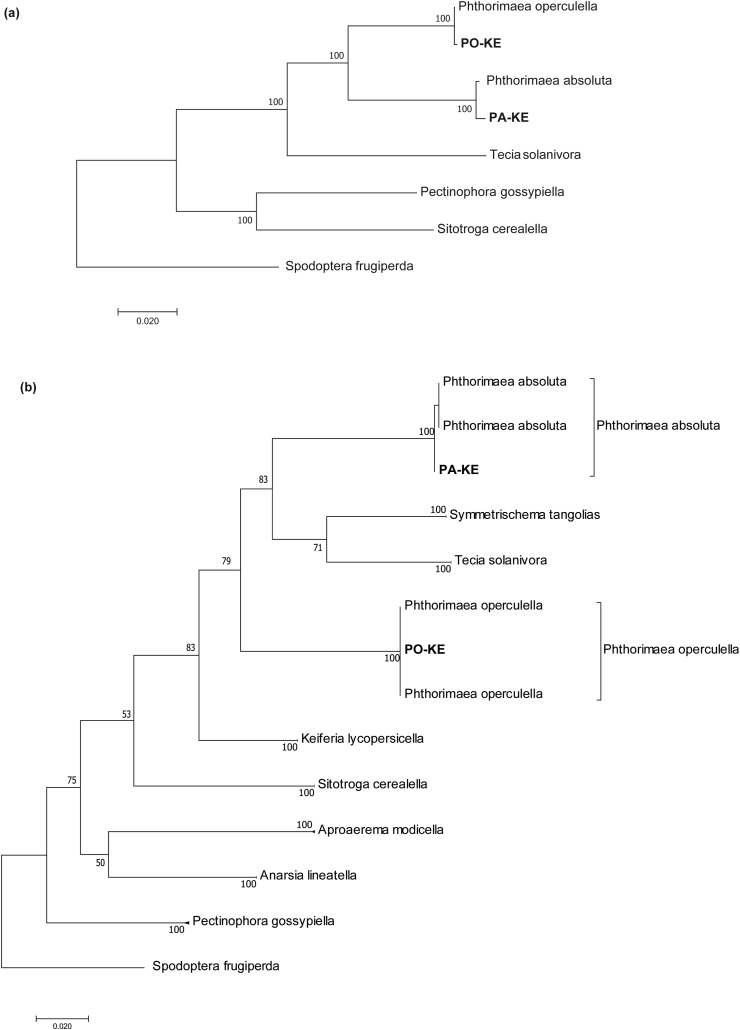
Maximum likelihood tree showing the phylogenetic clustering within the family Gelechiidae, using (a) the complete complement of the 13 mitochondrial protein-coding genes, (b) A 593-bp alignment of cytochrome *c* oxidase subunit 1 (COI) sequences. PO-KE - *Phthorimaea operculella* from this study; PA-KE - *Phthorimaea absoluta* from this study. Values represent nodal support calculated from 1000 bootstrap replicates *Spodoptera frugiperda* (Noctuidae) was used as an outgroup.

A Maximum Likelihood tree was built using the 13 PCGs obtained from the samples in this study combined with PCGs of the representative sequences available in GenBank to assess the phylogenetic structure of the *Phthorimaea* mitogenomes in relation to other members of the Gelechiidae family (Fig. 4a). The tree topology indicated that the *Phthorimaea* mitogenomes from this study formed a monophyletic cluster with the American potato tuber moth *T*. *solanivora*. The phylogeny based on the mitochondrial cytochrome oxidase I (COI) gene further showed the potato tuber moth, *P. operculella*, the tomato leafminer *P. absoluta*, the American potato tuber moth *T*. *solanivora* and the Guatemalan potato tuber moth *S*. *tangolais* formed a monophyletic cluster (Fig. 4b).

**Fig. 5:**
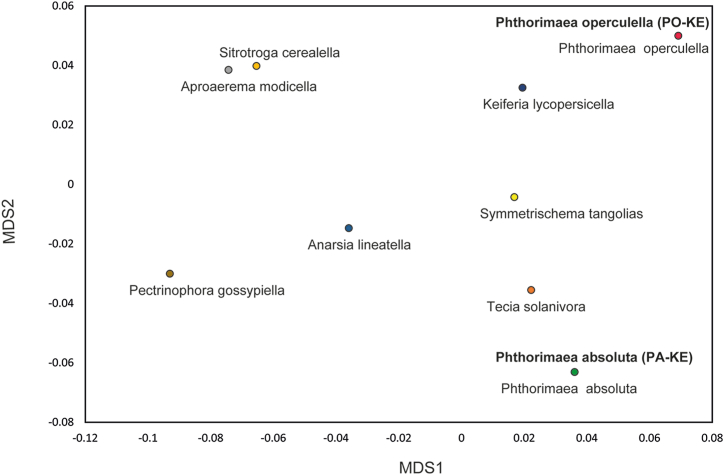
Inter-specific pairwise distances (K2P) in 9 species of the family Gelechiidae based on cytochrome *c* oxidase 1 (COI) sequences. Names in bold font represent samples from this study.

Genetic distance analyses for the *Phthorimaea* species included two new and 195 publicly available sequences for *P. absoluta*, two new and 28 publicly available sequences for *P. operculella* and 95 publicly available Gelechiidae sequences. For *P. absoluta* and *P. operculella*, no difference was found between the new sequences and the sequences available on GenBank. Interspecific genetic distances showed that *P. operculella* and *Pectinophora gossypiella* were the most diverged species (18.5%), while *P*. *absoluta* was most separated from *S*. *cerealella* (16.4%) (Table 2). The least divergence was seen between *P. operculella* and *K*. *lycopersicella* (6.2%). The PCoA plot based on genetic distances among all Gelechiidae showed an overlap of the new and publicly available *Phthorimaea* species (Fig. 5).

**Table 2:** Interspecific mean uncorrected p-distances (%) for the Gelechiidae family. Distances were calculated based on a 593 bp alignment of 322 new and publicly available sequences.

		Interspecific genetic divergence (%)										
		1	2	3	4	5	6	7	8	9	10	11
1	*Aproaerema modicella* (GenBank)	–										
2	*Sitotroga cerealella* (GenBank)	11.5	–									
3	*Phthorimaea absoluta* (GenBank)	15.9	16.4	–								
4	*Phthorimaea absoluta* (This study)	15.9	16.4	0.0	–							
5	*Tecia solanivora* (GenBank)	16.4	14.9	11.6	11.6	–						
6	*Symmetrischema tangolias* (GenBank)	14.3	13.0	11.2	11.2	8.9	–					
7	*Keiferia lycopersicella* (GenBank)	11.5	12.5	10.9	10.9	11.3	8.4	–				
8	*Phthorimaea operculella* (This study)	15.8	16.1	12.4	12.4	13.8	11.2	6.2	–			
9	*Phthorimaea operculella* (GenBank)	15.8	16.1	12.4	12.4	13.8	11.2	6.2	0.0	–		
10	*Pectinophora gossypiella* (GenBank)	11.7	13.1	15.0	15.0	15.9	14.6	13.7	18.5	18.5	–	
11	*Anarsia lineatella* (GenBank)	12.0	14.9	12.9	12.9	14.3	12.4	10.9	14.4	14.4	11.4	–

## 6 Habitat suitability and niche overlap

### 6.1 Predicted distribution

**Fig. 6:**
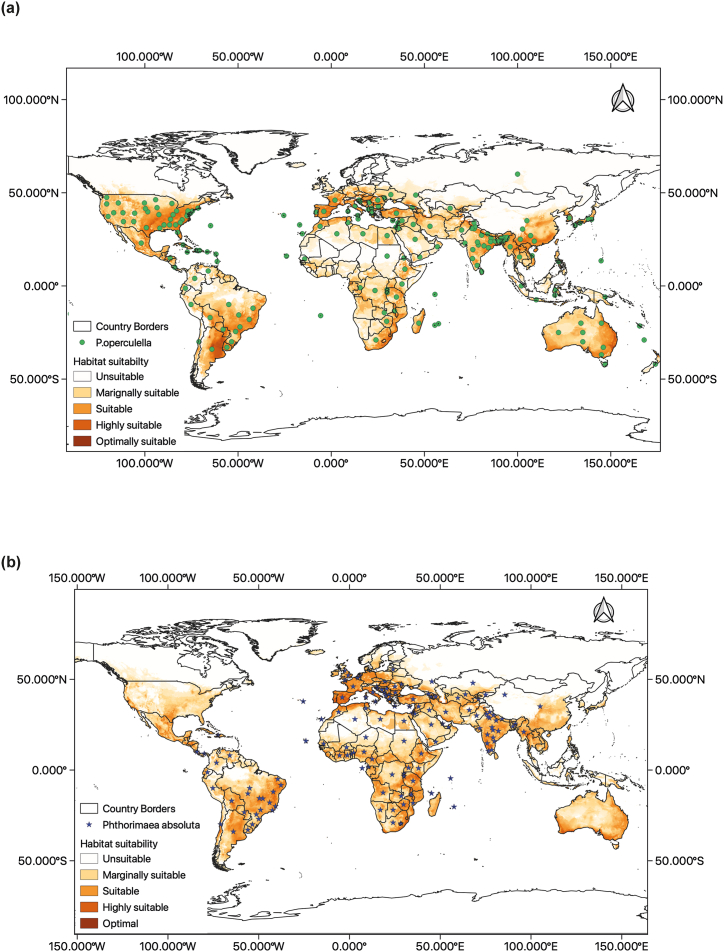
Two-model consensus (BIOCLIM and MaxEnt) of the global potential current distribution of (a) *Phthorimaea operculella* and (b) *Phthorimaea absoluta*. The map was generated using the World Geodetic System 1984 (WGS84) projection.

The predicted current distribution of *P. operculella* showed marginal to optimal suitability of the pest in all countries where the pest has been reported as countries where the pest has not been reported, such as Canada, the United Kingdom, some countries in southern Africa and most countries in Central and Western Africa (Fig. 6a). The predicted current distribution of *P. absoluta*, showed marginal to optimal suitability of the pest in all countries where the pest has been reported. In the countries where *P. absoluta* has not been reported, North America showed suitable to marginal suitability for the pest, while Australia showed regions of marginal to high suitability for the pest (Fig. 6b). The jackknife test for variable importance showed that the temperature-dependent variables had the highest permutation importance to the model for both species with annual mean temperature having the highest importance (Fig. S1).

### 6.2 Predicted niche overlap

**Fig. 7:**
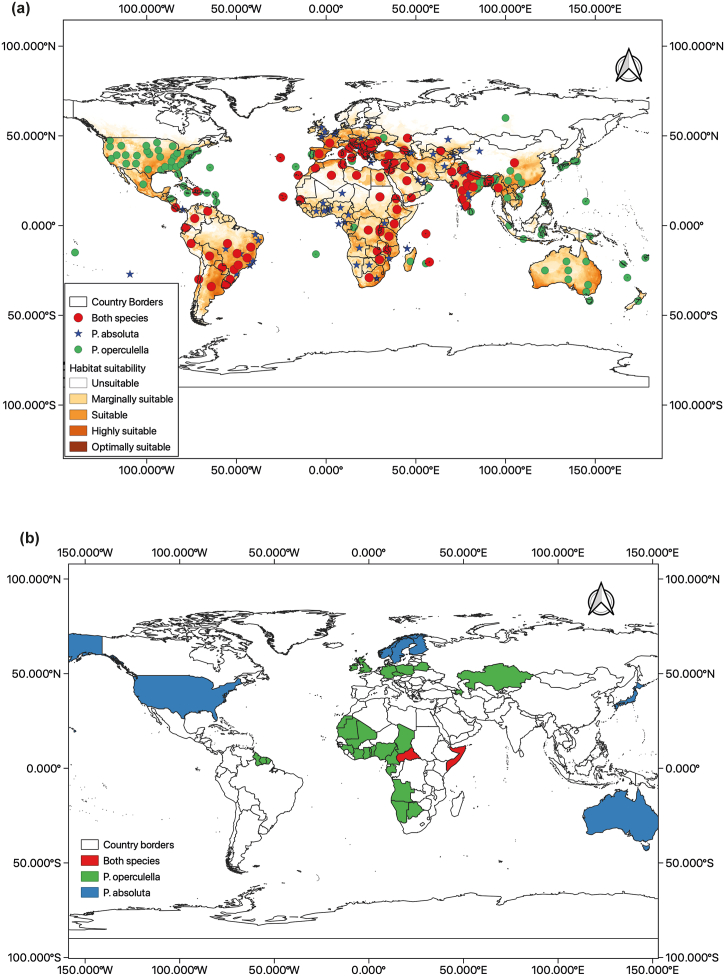
Niche overlap of *Phthorimaea operculella* and *Phthorimaea operculella* showing (a)Two-model consensus (BIOCLIM and MaxEnt) of the combined potential current distribution of *Phthorimaea operculella* and *Phthorimaea operculella* (b) countries with predicted suitability for *Phthorimaea operculella* and *Phthorimaea operculella* with no reports of either pest. The maps were generated using the World Geodetic System 1984 (WGS84) projection.

The predicted distribution of the two *Phthorimaea* species obtained from the two-model consensus showed distribution in large areas of Africa, the Americas, Asia and Australia. The overlap of the prediction with the current global distribution of *P. absoluta* revealed regions of North America and Australia where the presence of *P. absoluta* has not been reported showed high to optimal suitability for the pest (Fig. 7a). The overlap of the prediction with the current global distribution of *P. operculella* also indicated areas where the presence of *P. operculella* has not been reported, like most western and central African countries, have marginal to high suitability for the pest (Fig. 7b).

## 7 Discussion

The potato tuber moth (*P*. *operculella*) and the tomato leaf miner (*P. absoluta*) are devastating pests of potato and tomato, respectively [[68], [69], [70], [71]]. Both species can complete their lifecycles on either crop [4,11,72] thus presenting a significant threat to solanaceous crop production. While *P. operculella* has been reported in most potato-growing areas of the world, there are no documented reports of *P. absoluta* in North America and Australia [50,52,54]. Our study provides insight into the phylogenetic relationship between the *Phthorimaea* species within the Gelechiidae family, the spatial distribution of the two *Phthorimaea* species and areas of potential invasion of the pests.

There has been ambiguity in the classification of members of the Gelechiidae family, as members have been classified and reclassified on the basis of their morphology and are prime candidates for in-depth phylogenomics to resolve their taxonomic ambiguity [16]. Previous classification changes were not accompanied by in-depth analysis as such a concrete argument and framework for the placement of the species is required, as seen in the current classification of *P. absoluta* in the *Phthorimaea* genus through a cladistic morphological approach [16]. Individually, the mitochondrial genome of the two species has been assembled [73,74]. However, a comparative look at these closely related species has not been assessed. The comparison of the complete mitogenomes of *P. operculella* and *P*. *absoluta* in this study, showed common and unique structural features, including the proportion and size of PCGs, tRNAs and IGSs. The size and structure of both mitogenomes obtained in this study were identical to those of other Gelechiids in gene order and gene orientation and were identical to the hypothesised metazoan ancestral mitogenome arrangement [67,75]. This was also seen in the comparison of members of the family Trigonidiidae [76], Triozidae [77], Psyllidae [78] and Gryllotalpidae [79]. In both species, the start codons of most PCGs were ATG, ATT, and ATA, typical for arthropod mitochondrial PCGs [76], except for COI, ND4, ND4l and ND1, which had CGA and CAT start codons. The CGA start codon in the COI gene was also observed in *Spodoptera frugiperda* [80]. There were no variations in the length of most PCGs in both species except for COI, ATP8, ND5 and ND1, which had slight variations (between 3 and 6 bp). It was observed that between ND4 and ND4l, *P*. *absoluta* had a 2 bp IGS region while *P. operculella* had a 1 bp IGS. The presence of a 7 bp overlap between ND4 and ND4l is a common feature in insects [81], and a mature bicistronic transcript can be formed by the two genes [82]. However, this compact IGS in the *Phthorimaea* species may not affect the transcription into a mature bicistronic mRNA in both species, as seen in *D. fascipes* and *D. furumagiensis*, although, exceptions to the 7 bp overlap have been seen, for example in *Spodoptera frugiperda* [80]. The highest number of SNPs was seen in COI, while the least was in ATP8. However, ATP8 had the highest percentage of non-synonymous substitutions resulting in amino acid changes, while COI had the least percentage. This is because the COI gene is highly conserved within the Lepidopteran order. Buhay [83] surmised COI to be a very conserved gene and changes in nucleotides are usually synonymous and do not lead to amino acid changes. The genetic diversity of *P. operculella* and *P*. *absoluta* showed a low genetic divergence (6.2%) based on the COI gene. Since each species can complete its life cycle on the principal host plant of the other [2], future insights into the choice of host plants for both insects could be obtained from the investigation of the whole genomes of specimens collected from these insects. Furthermore, the pattern of divergence of *P. operculella*, *P*. *absoluta, K. lycopersicella, T. solanivora*, and *S. tangolias* relative to the other Gelichiids was interesting as the species in the cluster had a close genetic similarity with p-distances ranging between 6.2% and 12.4%. However, this cluster was genetically distinct from the other species, with p-distances higher than 14.6%. Although a universal threshold for using p-distances based on DNA barcodes to delimit species cannot be established [75,84], this result warrants a closer look into the taxonomic status of Gelechiids.

Our overall results present a hypothesis showing the diversity within the Gelechiidae family and propose that *P. operculella*, *P*. *absoluta, K. lycopersicella, T. solanivora*, and *S. tangolias* constitute members of the same genus. However, this theory is based on the analyses of a short region of the COI gene (593 bp), and species delineation should not be made solely on this region [79]. Therefore, utilizing multiple genes to differentiate species may provide a clearer picture of the genetic structure of insect species [71]. For instance, ND5 and COI gene regions have been applied in studies on the genetic diversity of flies [85], CytB has been used to investigate the population structure in *Athetis lepigone* [86], and CYTB, ND5, ND4, ND6 and ATP6 were the most polymorphic regions of two Saturniidae species [87]. Furthermore, mitochondrial DNA is limited in detecting hybridisation and Introgression events. Therefore, analysis of nuclear sequence data may resolve the species structure. Consequently, we recommend further investigation involving additional cladistic analysis of morphological, genetic and ecological data in order to make definite conclusions. Nevertheless, our results indicate an ambiguous diversity in the Gelechiidae family and reiterates further studies into the relationship between insect haplogroups and host plant [70].

The genetic similarities of the two *Phthorimaea* species and their ability to survive on the primary host plant of either species may inform their high rate of spread and invasive capacities. Species distribution and predictive modelling for both species have been investigated using various models; Boosted Regression Trees, Cellular automation and CLIMEX [6,33,34,88]. However, considering the overlap in host plants of both pests, a combined distribution model for both pests has not been investigated. Our results highlight the distribution of *P. absoluta* and *P. operculella* and reveal that most potato and tomato-producing areas in Africa, the Americas, Australia, Asia and Europe are suitable for both pests based on the similarities in the overall permutation importance of the temperature variables of both species identified in the model as well as the optimal range for development of both species which has been noted to range from 20 to 30 °C [6,89]. North America had areas of marginal to high suitability for *P. absoluta* establishment, likewise, Australia and some parts of Europe showed marginal to high suitability for the pest, although *P. absoluta* has not been reported in these areas. We also observed the areas of marginal to high suitability for *P. operculella* in large potato-producing regions of Europe, central, west and southern Africa where the pest has not been reported. Although the pests have not been reported in these areas, their presence cannot be ruled out. Therefore, intensive surveys would be critical to determine the presence or establishment of the pest in these areas. Our results agree with the studies on the predicted distribution of *P. absoluta* in Africa and Asia [6,88] and the global studies on the potential spread of potato tuber worms *P. operculella* [34,90], *T. solanivora* [91] and *S. tangolias* [92], which showed moderate to high risk of establishment of these pests in Europe, central, west and southern Africa. The overlay of the individual models for both species showed a significant niche overlap. The overlap concurred with areas where both pests have been reported and highlighted countries where neither pest has been reported but is suitable for the establishment of both pests. This presents a case for increased monitoring, surveillance and strict phytosanitary measures in these countries. Detection of *P. operculella* larval infestation of potato tubers difficult. Still, the presence of the pest can be confirmed in shipments by observing adult moths in storage or silk cocoons on the tuber surface [31]. These signs during potato shipping and storage call for immediate phytosanitary measures. Strict phytosanitary procedures have been recommended for countries where the pest has not been established therefore, surveillance and pest status verification for the two *Phthorimaea* species should be carried out in key production areas [31].

Finally, elucidating the genetic diversity and potential distribution of Gelechiids can increase the understanding of the invasion and dispersal dynamics of the species of economic importance within this family. This aspect is key in developing sustainable control and preventive measures for *Phthorimaea* species due to the evidence of the high potential for invasion of *P. absoluta*. Furthermore, elucidating the genetic diversity within the Gelechiids using nuclear biparentally transferred genes, for example, will contribute to identifying invasion histories of the pests and, in addition to predictive modelling, aid in identifying potential cross-border invasions. Our projection of the distribution of the *Phthorimea* species and their niche overlap will guide vector monitoring strategies in potato and tomato-producing areas with high propensity for the pests’ establishment. We demonstrated the genetic diversity and predicted the potential distribution/niche overlap between the potato tuber worm (*P. operculella*) and the tomato leafminer (*P. absoluta*). These results present a clear case for stricter phytosanitary considerations and further research into the genetic constitution and taxonomic harmonization of the members of the Gelechiidae family. This would be key in informing strategies for integrated management systems for solanaceous crop production.

## Data availability statement

All sequences generated in this study were deposited in GenBank database (www.ncbi.nlm.nih.gov/genbank) under the BioProject: PRJNA902348, and the raster layers of the models generated in this study were deposited in dryad DOI https://doi.org/10.5061/dryad.3j9kd51nw.

## CRediT authorship contribution statement

**Inusa Jacob Ajene:** Writing – review & editing, Writing – original draft, Visualization, Validation, Software, Investigation, Formal analysis, Data curation, Conceptualization, Methodology. **Helen Heya:** Writing – review & editing, Data curation. **Fathiya Mbarak Khamis:** Writing – review & editing, Validation, Resources, Project administration, Funding acquisition, Data curation, Conceptualization.

## Declaration of competing interest

The authors declare that they have no known competing financial interests or personal relationships that could have appeared to influence the work reported in this paper.
