# Supplementary material for: Evaluating the mitochondrial genomic diversity, global distribution and niche overlap of two invasive Phthorimaea species
Source: Heliyon. 2024 Apr 4;10(7):e29010. doi: 10.1016/j.heliyon.2024.e29010 (PMC11015427; doi:10.1016/j.heliyon.2024.e29010)
Supplement: Multimedia component 1 [file mmc1.docx]

**Supplementary materials**

**Table S1**. List of the five complete mitochondrial sequences used in the phylogenetic reconstruction of the family Gelechiidae(Lepidoptera) with common name, GenBank accession numbers and bibliographic references. *Spodoptera frugiperda* (Lepidoptera: Noctuidae) was used as an outgroup.

| **Species** | **Family** | **Common name** | **GenBank** | **Reference** | **Size (bp)** |
| --- | --- | --- | --- | --- | --- |
| *Pectinophora gossypiella* | Gelechiidae | Pink bollworm | NC_065403.1 | Zhao et al., 2022 | 15202 |
| *Phthorimaea absoluta* | Gelechiidae | Tomato leaf miner | Upon acceptance | This study | 15292 |
| *Phthorimaea absoluta* | Gelechiidae | Tomato leaf miner | NC_050874 | Zhang et al., 2019 | 15290 |
| *Phthorimaea operculella* | Gelechiidae | Potato tuber moth | Upon acceptance | This study | 15267 |
| *Phthorimaea operculella* | Gelechiidae | Potato tuber moth | NC_057501 | Kim et al., 2020 | 15263 |
| *Sitotroga cerealella* | Gelechiidae | Grain moth | NC_041123.1 | Yuan et al., 2019 | 15312 |
| *Spodoptera frugiperda* | Noctuiidae | Fall armyworm | NC_027836.1 | Liu, 2014 | 15365 |
| *Tecia solanivora* | Gelechiidae | Guatemalan potato tuber moth | NC_029386.1 | Ramirez-Rios et al., 2016 | 15251 |

**Table S2.** List of publicly available and new COI sequences (n = 322) from members of the family Gelechiidae used for the construction of the maximum likelihood tree and estimates of genetic distances and genetic diversity.

| **Species** | **Genbank Accession** | **Source Country** |
| --- | --- | --- |
| *Pthorimaea absoluta PA-KE* | Upon acceptance | Kenya |
| *Pthorimaea operculella PO-KE* | Upon acceptance | Kenya |
| *Anarsia lineatella* | KM572249 | Finland |
|  | KM573689 | Austria |
|  | JF859823 | Italy |
|  | GU706949 | Germany |
|  | HQ563679 | Germany |
|  | HQ563768 | Germany |
|  | HQ955190 | Germany |
|  | JN264932 | Denmark |
|  | JN264937 | Italy |
|  | JN264938 | Italy |
|  | MN804974 | Austria |
|  | MN803601 | Germany |
|  | MN803849 | Germany |
|  | MN803910 | Germany |
|  | KM572596 | Finland |
|  | GU706948 | Germany |
| *Aproaerema modicella* | MN525169 | India |
|  | MN525164 | India |
|  | MN525163 | India |
|  | MN525162 | India |
|  | MN525168 | India |
|  | MN525167 | India |
|  | MN525165 | India |
| *Keiferia lycopersicella* | KJ657678 | - |
|  | MT021739 | USA |
|  | MT021738 | USA |
|  | MT021737 | USA |
|  | KY442264 | USA |
|  | JN305836 | USA |
|  | MT021741 | USA |
|  | MT021740 | USA |
| *Pectinophora gossypiella* | MH998203 | India |
|  | MH998202 | India |
|  | MK371446 | India |
|  | MZ049693 | India |
|  | MH998156 | India |
|  | MH998157 | India |
|  | MH998158 | India |
|  | MH998159 | India |
|  | MH998160 | India |
|  | MH998161 | India |
|  | MH998162 | India |
|  | MH998164 | India |
|  | MH998165 | India |
|  | MH998166 | India |
|  | MH998167 | India |
|  | MH998168 | India |
|  | MH998169 | India |
|  | MH998170 | India |
|  | MH998171 | India |
|  | MH998172 | India |
|  | MH998173 | India |
|  | MH998174 | India |
|  | MH998175 | India |
|  | MH998176 | India |
|  | MH998177 | India |
|  | MH998178 | India |
|  | MH998179 | India |
|  | MH998180 | India |
|  | MH998181 | India |
|  | MH998182 | India |
|  | MH998183 | India |
|  | MH998184 | India |
|  | MH998185 | India |
|  | MH998186 | India |
|  | MH998187 | India |
|  | MH998188 | India |
|  | MH998189 | India |
|  | MH998190 | India |
|  | MH998191 | India |
|  | MH998192 | India |
|  | MH998193 | India |
|  | MH998194 | India |
|  | MH998195 | India |
|  | MH998196 | India |
|  | MH998197 | India |
|  | MH998198 | India |
|  | MH998199 | India |
|  | MH998201 | India |
|  | MH998200 | India |
|  | MH998163 | India |
|  | KM268793 | India |
| *Phthorimaea operculella* | KX443099 | - |
|  | KX443100 | - |
|  | KX443101 | - |
|  | KX443102 | - |
|  | KF387868 | Australia |
|  | KF387969 | Australia |
|  | KF388684 | Australia |
|  | KF390269 | Australia |
|  | KY442082 | USA |
|  | MF121865 | Kenya |
|  | MF121866 | Kenya |
|  | MF121867 | Kenya |
|  | MF121868 | Kenya |
|  | MF121869 | Kenya |
|  | MF121870 | Kenya |
|  | MF121871 | Kenya |
|  | MF121872 | Kenya |
|  | MF121874 | Kenya |
|  | MF121875 | Kenya |
|  | MF121876 | Kenya |
|  | MF121878 | Kenya |
|  | MF121879 | Kenya |
|  | MF121880 | Kenya |
|  | MF121882 | Kenya |
|  | MF121881 | Kenya |
|  | MF121873 | Kenya |
|  | KX443098 | - |
|  | MN804103 | Italy |
| *Sitotroga cerealella* | KY492361 | India |
|  | MW452547 | Egypt |
| *Spodoptera frugiperda* | NC_027836 | - |
| *Symmetrischema tangolias* | KY951809 | Australia |
|  | KX443107 | - |
|  | KX443106 | - |
|  | KX443105 | - |
|  | KX443104 | - |
|  | KX443103 | - |
|  | KY951760 | Australia |
|  | KY442097 | Australia |
| *Tecia solanivora* | KY442260 | Costa Rica |
|  | MN804985 | Spain |
|  | KY818717 | - |
| *Pthorimaea absoluta* | KU565720 | Kenya |
|  | KU565719 | Kenya |
|  | KU565522 | Kenya |
|  | KU565523 | Kenya |
|  | KU565524 | Kenya |
|  | KU565525 | Kenya |
|  | KU565527 | Kenya |
|  | KU565526 | Kenya |
|  | KU565528 | Kenya |
|  | KU565529 | Kenya |
|  | KU565532 | Peru |
|  | KU565533 | Peru |
|  | KU565534 | Peru |
|  | KU565536 | Kenya |
|  | KU565537 | Kenya |
|  | KU565538 | Kenya |
|  | KU565539 | Kenya |
|  | KU565541 | Kenya |
|  | KU565542 | Kenya |
|  | KU565543 | Kenya |
|  | KU565545 | Kenya |
|  | KU565544 | Kenya |
|  | KU565546 | Kenya |
|  | KU565547 | Kenya |
|  | KU565548 | Kenya |
|  | KU565549 | Kenya |
|  | KU565550 | Kenya |
|  | KU565551 | Kenya |
|  | KU565555 | Kenya |
|  | KU565554 | Kenya |
|  | KU565553 | Kenya |
|  | KU565552 | Kenya |
|  | KU565557 | Kenya |
|  | KU565556 | Kenya |
|  | KU565558 | Kenya |
|  | KU565561 | Kenya |
|  | KU565560 | Kenya |
|  | KU565559 | Kenya |
|  | KU565565 | Kenya |
|  | KU565564 | Kenya |
|  | KU565563 | Kenya |
|  | KU565562 | Kenya |
|  | KU565566 | Kenya |
|  | KU565570 | Kenya |
|  | KU565569 | Kenya |
|  | KU565568 | Kenya |
|  | KU565567 | Kenya |
|  | KU565573 | Kenya |
|  | KU565572 | Kenya |
|  | KU565571 | Kenya |
|  | KU565576 | Kenya |
|  | KU565575 | Kenya |
|  | KU565574 | Kenya |
|  | KU565579 | Kenya |
|  | KU565578 | Kenya |
|  | KU565577 | Kenya |
|  | KU565580 | Kenya |
|  | KU565582 | Kenya |
|  | KU565583 | Kenya |
|  | KU565584 | Kenya |
|  | KU565585 | Kenya |
|  | KU565586 | Kenya |
|  | KU565588 | Kenya |
|  | KU565587 | Kenya |
|  | KU565589 | Kenya |
|  | KU565591 | Kenya |
|  | KU565590 | Kenya |
|  | KU565592 | Kenya |
|  | KU565595 | Kenya |
|  | KU565594 | Kenya |
|  | KU565593 | Kenya |
|  | KU565596 | Kenya |
|  | KU565597 | Kenya |
|  | KU565602 | Kenya |
|  | KU565601 | Kenya |
|  | KU565600 | Kenya |
|  | KU565599 | Kenya |
|  | KU565598 | Kenya |
|  | KU565604 | Kenya |
|  | KU565603 | Kenya |
|  | KU565605 | Kenya |
|  | KU565606 | Kenya |
|  | KU565607 | Kenya |
|  | KU565608 | Kenya |
|  | KU565609 | Kenya |
|  | KU565610 | Kenya |
|  | KU565611 | Kenya |
|  | KU565612 | Kenya |
|  | KU565613 | Kenya |
|  | KU565614 | Kenya |
|  | KU565615 | Kenya |
|  | KU565616 | Kenya |
|  | KU565617 | Kenya |
|  | KU565618 | Kenya |
|  | KU565619 | Kenya |
|  | KU565620 | Kenya |
|  | KU565621 | Kenya |
|  | KU565622 | Kenya |
|  | KU565623 | Kenya |
|  | KU565624 | Kenya |
|  | KU565625 | Kenya |
|  | KU565626 | Kenya |
|  | KU565627 | Kenya |
|  | KU565628 | Kenya |
|  | KU565629 | Kenya |
|  | KU565630 | Kenya |
|  | KU565631 | Kenya |
|  | KU565632 | Kenya |
|  | KU565633 | Kenya |
|  | KU565634 | Kenya |
|  | KU565635 | Kenya |
|  | KU565636 | Kenya |
|  | KU565637 | Kenya |
|  | KU565638 | Kenya |
|  | KU565639 | Kenya |
|  | KU565640 | Kenya |
|  | KU565641 | Kenya |
|  | KU565642 | Kenya |
|  | KU565643 | Kenya |
|  | KU565644 | Kenya |
|  | KU565645 | Kenya |
|  | KU565646 | Kenya |
|  | KU565647 | Kenya |
|  | KU565648 | Kenya |
|  | KU565649 | Kenya |
|  | KU565650 | Kenya |
|  | KU565651 | Uganda |
|  | KU565652 | Uganda |
|  | KU565653 | Uganda |
|  | KU565654 | Uganda |
|  | KU565655 | Uganda |
|  | KU565656 | Tanzania |
|  | KU565657 | Tanzania |
|  | KU565658 | Tanzania |
|  | KU565660 | Tanzania |
|  | KU565659 | Tanzania |
|  | KU565661 | Kenya |
|  | KU565662 | Kenya |
|  | KU565663 | Kenya |
|  | KU565664 | Kenya |
|  | KU565665 | Kenya |
|  | KU565666 | Kenya |
|  | KU565667 | Kenya |
|  | KU565668 | Kenya |
|  | KU565669 | Kenya |
|  | KU565670 | Kenya |
|  | KU565671 | Kenya |
|  | KU565672 | Kenya |
|  | KU565673 | Kenya |
|  | KU565674 | Kenya |
|  | KU565675 | Kenya |
|  | KU565676 | Kenya |
|  | KU565677 | Kenya |
|  | KU565678 | Kenya |
|  | KU565679 | Kenya |
|  | KU565680 | Kenya |
|  | KU565681 | Kenya |
|  | KU565682 | Kenya |
|  | KU565683 | Kenya |
|  | KU565686 | Tanzania |
|  | KU565685 | Kenya |
|  | KU565684 | Kenya |
|  | KU565687 | Tanzania |
|  | KU565688 | Tanzania |
|  | KU565689 | Tanzania |
|  | KU565690 | Tanzania |
|  | KU565691 | Kenya |
|  | KU565692 | Kenya |
|  | KU565693 | Kenya |
|  | KU565694 | Kenya |
|  | KU565695 | Kenya |
|  | KU565696 | Kenya |
|  | KU565697 | Kenya |
|  | KU565698 | Kenya |
|  | KU565699 | Kenya |
|  | KU565700 | Kenya |
|  | KU565701 | Kenya |
|  | KU565702 | Kenya |
|  | KU565703 | Kenya |
|  | KU565704 | Kenya |
|  | KU565705 | Kenya |
|  | KU565706 | Kenya |
|  | KU565707 | Kenya |
|  | KU565708 | Kenya |
|  | KU565709 | Kenya |

**Table S3.** Predictor bioclimatic variables used for modelling the ecological niche for *Phthorimaea absoluta* and *Phthorimaea operculella*. Data was sourced from the WorldClim database accessed on October 2022.

| **Variable** | **Code** | **Units** |
| --- | --- | --- |
| Annual mean temperature  Mean diurnal range  Isothermality  Temperature Seasonality  Max temperature of warmest month  Min temperature of coldest month quarter  Temperature annual range  Mean temperature of wettest quarter  Mean temperature of driest quarter  Mean temperature of warmest quarter  Mean temperature of coldest quarter  Annual precipitation  Precipitation of wettest month  Precipitation of driest month  Precipitation seasonality (coefficient of variation)  Precipitation of wettest quarter  Precipitation of driest quarter  Precipitation of warmest quarter  Precipitation of coldest quarter | Bio 1  Bio 2  Bio 3  Bio 4  Bio 5  Bio 6  Bio 7  Bio 8  Bio 9  Bio 10  Bio 11  Bio 12  Bio 13  Bio 14  Bio 15  Bio 16  Bio 17  Bio 18  Bio19 | ^o^C  ^o^C  ^o^C  ^o^C  ^o^C  ^o^C  ^o^C  ^o^C  ^o^C  ^o^C  ^o^C  mm  mm  mm  mm  mm  mm  mm  mm |


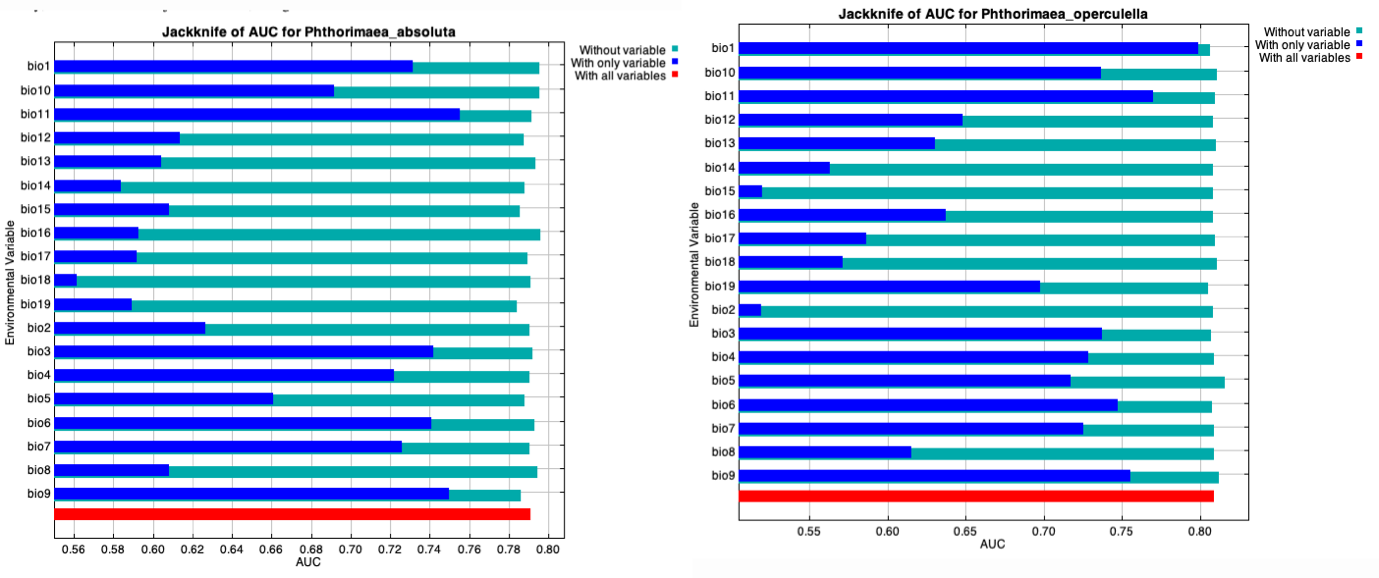


**Figure S1.** Jacknife test of the AUC, for *Phthorimaea absoluta* and *Phthorimaea operculella*.

**Data Accessibility**

All sequences generated in this study were deposited in GenBank database ([www.ncbi.nlm.nih.gov/genbank](http://www.ncbi.nlm.nih.gov/genbank)) under the BioProject: PRJNA902348 and the raster layers of the models generated in this study were deposited in dryad DOI <https://doi.org/10.5061/dryad.3j9kd51nw>.
